# Why Health-enhancing Nudges Fail

**DOI:** 10.1007/s10728-023-00459-7

**Published:** 2023-07-21

**Authors:** Thomas Schramme

**Affiliations:** Department of Philosophy, University of Liverpool, Gillian Howie House Mulberry Street, Liverpool, L69 7SH UK

**Keywords:** Nudging, Health, Sunstein, Paternalism, Public health

## Abstract

Nudges are means to influence the will formation of people to make specific choices more likely. My focus is on nudges that are supposed to improve the health condition of individuals and populations over and above the direct prevention of disease. I point out epistemic and moral problems with these types of nudges, which lead to my conclusion that health-enhancing nudges fail. They fail because we cannot know which choices enhance individual health—properly understood in a holistic way—and because health-enhancing nudges are often themselves bad for our health. They can be bad for our health because they assume inferior agency in their targets and accordingly regularly lead to appropriate resentment and anger—strong emotions which go along with an increased risk of health impairments. Briefly, health-enhancing nudges fail because they are based on persistent ignorance and on a presumptuous attitude.

## Introduction

The topic of my paper is a specific type of nudge. Nudges are means to influence the will formation of people so that specific choices are more likely to be the outcome of decision processes. The specific nudges I discuss are health-enhancing nudges. The aim of these nudges is to improve the health condition of individuals and populations. As will be shown in this paper, what exactly that means is not quite clear, simply because the concept of health is not straightforward. Although I will not have enough space to thoroughly discuss the relevant concept, I will be able to say enough to gain a proper footing for assessing health-enhancing nudges. My conclusion is that health-enhancing nudges fail, at least when introduced by governmental agencies. They fail because we cannot know which choices reliably enhance individual health—properly understood in a holistic way—and because health-enhancing nudges can themselves be bad for our health. They are prone to be bad for our health because they assume inferior agency in their targets and accordingly regularly lead to appropriate resentment and anger—strong emotions which go along with an increased risk of health impairments. Succinctly put, health-enhancing nudges fail because they are based on persistent ignorance and on a presumptuous attitude.

Critically assessing nudges can be a frustrating task. Even pinning down the object of discussion is taxing; it is a moving target. There is no agreed definition of nudges. It is furthermore difficult to assess nudges as such, because they are too diverse. We should therefore focus on specific practices when assessing nudges.^1^ In this paper, I discuss health-enhancing nudges, specifically the example of nudging people towards healthier food options. My topic is sufficiently restricted, I believe, to allow conclusive assessment.

My examination proceeds as follows: In the next, second, section I introduce my understanding of nudges and specifically of health-enhancing nudges. The third section introduces epistemic problems for planners. Health, properly understood, is a holistic term, including more than organismic functioning. In addition, enhancing health, even if deemed possible via nudges, has a different normative status from tackling occurrent disease. Having shown that typical health-enhancing nudges will be unlikely to succeed, I add, in the fourth section, that they can easily be harmful, because they lead to legitimate resentment and anger in many people. It is resented that nudges tread on individuals’ agency and assume better ability in relation to complex and deeply individual choices. Since being a chooser of one’s own is an important element of the good life for human beings, health-enhancing nudges can, in a slogan, be bad for our health.

## Health-enhancing Nudges and Their Justification

Nudges, as I understand them, are techniques that intentionally design the choice environment of people in a way that makes specific aspects more salient to them and hence aim at making certain choices more likely.^2^ Note that this interpretation allows for both paternalistic and non-paternalistic nudges. The latter nudges usually aim to reduce so-called negative externalities—costs to others—not to enhance the welfare of the chooser.^3^ This is important, because health-enhancing nudges can also both be seen as paternalistic and as intended to reduce health-related costs to others. After all, ill health usually causes costs to others in virtue of the requirement to maintain a system of health care, financed through taxes or insurances. There are of course specific problems to do with paternalistic practices as such. I ignore these issues; the specific motivations of health-enhancing nudges are not my main concern in this paper but their effectiveness.

Examples of health-related nudges are the layout of food options, for instance in supermarkets, and emotionally charged information, such as graphic images on cigarette packs. There are many other mechanisms that make certain choices more likely, for instance gamification and ambient design. Gamification can be used as an incentive to perform certain tasks, say, going for a run. Ambient design, such as emotionally charged lighting, can lead to a more relaxed mood of patients in a hospital. I will not discuss whether these instruments are nudges. After all, my main concern is not conceptual analysis. The types of examples I discuss are nudges according to any standard interpretation.

Unfortunately, Cass Sunstein, who did a lot to promote nudges in numerous publications and as a member of the Obama administration from 2009 to 2012, is not very helpful when it comes to the concept of nudges. He has used various definitions in different publications. A recent definition is: “A *nudge* is defined as an intervention, from either private or public institutions, that affects people’s behavior while fully maintaining their freedom of choice. A GPS device is a canonical example. It tells you what route to take and thus helps you get where you want to go—but you specify the destination, and you can reject its advice and take your own route if you prefer. A default rule is a nudge, so long as you can easily opt out. The same is true of warnings and disclosure of information” (Sunstein, [2020], 4). This definition is far too broad. A GPS device, for instance, does not intentionally make any choice more salient; it simply provides information in a neutral way, similar to sign-posts. It is true that a GPS can be set to follow particular preferences, for instance whether a driver wants to avoid certain types of roads or prefers to take the shortest route. But even if we would deem this type of preference-induced information an example of nudging, the GPS system as such would not represent the nudging mechanism. In general, warnings and providing information should not count as nudges (pace Sunstein). They do not make any choice more salient for a chooser, though of course based on their preferences facts partly determine their individual choice.^4^

Health nudges come in different types. An example is a reminder sent to a patient about a doctor’s appointment or about taking medicine. Such types of nudges aim at improving treatment of an ill patient or the prevention of (symptoms of) specific diseases, such as epilepsy, diabetes or blood clotting. Accordingly, these examples are health nudges in the sense of restoring or maintaining a minimal level of health, where minimal health is understood as absence of disease.

In contrast, the type of nudges I will discuss can be called health-enhancing nudges because they aim at improving health over and above the absence of disease. They aim at making people healthier—or fitter, to use a different, perhaps more adequate, term. I will use mainly examples of nudges for healthy lifestyles, especially regarding eating habits. To eat more nutritious and less fatty food, for instance, does not result in curing a disease or directly maintaining minimal health, although it might make it less likely for a person to fall ill in the future. Health-enhancing nudges are concerned with positive health or ideal health, not minimal health. I will argue that these types of nudges fail when introduced by a planning agency. They might fare better when personalised, but I raise some doubts in this respect as well.

Health-enhancing nudges are usually paternalistically motivated. That is, they aim at benefitting nudged persons by making choices more likely that are potentially against their current desires but nevertheless serve their interests. As mentioned, many health nudges can alternatively be deemed non-paternalistic, because they might aim at a benefit for others, for instance in virtue of reducing waste of prescribed medicine or scarce time of health care professionals by nudging people to take medication or observe appointments. Health nudges can therefore reduce health-care related costs to taxpayers or insured citizens.^5^

A common strategy to defend nudges is to point out that citizens themselves want them. More specifically, Sunstein and Thaler say that nudges “make choosers better off, *as judged by themselves*” (Thaler & Sunstein, [2008], 5 (emphasis in original)). If people indeed want to be supported by nudges in improving their choices, then this might justify state-induced health-enhancing nudges. What does it mean, exactly, that nudges are in congruence with what people want? It might mean (i) that people want to be nudged; (ii) that they endorse the values that nudges pursue, such as wealth, health, and happiness; (iii) that nudges are in line with individuals’ “deeper”, “latent” or “true” preferences (Scoccia, [2019], 80; Sugden, [2017], 116; cf. Desroches, 2020).

Regarding the first interpretation, it seems wrong to assume that people want to be nudged by governmental agencies, although we would need some more empirical findings to build a proper case in either direction (Sugden, [2017], 122; cf. Sunstein, 2016b, 116; Sunstein, [2018], 5). They might want to be nudged by themselves, or rather by nudging mechanisms set up by themselves. For instance, they might want to purchase wearables to nudge them to exercise.^6^ It might be said that people themselves agree that they are bad at making certain decisions, for instance health-related choices. Yet that would not back the claim that people endorse health-enhancing nudges. Individuals can maintain that there is a significance and value even in making bad decisions; and even if they have regrets about choices, that does not mean that they want others to nudge them in another direction. Accordingly, individuals can agree with the aims of nudges without agreeing with nudges being administered (cf. Glod, [2015], 606).

The second interpretation makes a fairly intuitive claim: that people want to be happy, healthy and wealthy. Nudges try to help them to achieve exactly that. But again, it is obvious that sharing a value does not imply sharing an agreement about the value of instruments that might help in achieving these overarching values. For instance, many people value personal relationships, but that does not mean that they are in favour of allocating a companion to lonely people.

The third interpretation makes assumptions about what individuals would choose or prefer if they were fully rational (Sunstein and Thaler 2003, 1162; cf. Grüne-Yanoff, [2012], 642). Nudges, according to this interpretation help people to choose what they really want or what is in their “best interest” (Sunstein and Thaler 2003, 1163), as opposed to what they choose in reality. It is aiming at an “as-if rationality” (Sunstein, [2014], 154). But surely this is not a helpful argument, because it is merely based on theoretical assumptions, framed in a particular model. Human rationality or interests do not point at particular choices, except if certain theoretical assumptions about relevant aims are introduced.^7^ It is actually ironic that behavioural economists would use this argument, because they started their own research paradigm from a critique of such an idealised model of choice.^8^

Still, at least in terms of health-enhancing nudges the required assumption about people’s goals seems easy enough to make: The relevant element of the good for human beings is health. This is also what people themselves say they want. Yet there is an epistemic problem regarding the relative value of health (White, [2016], 22 f.). How much do people value health? Are they, for instance, prepared to bear significant costs when pursuing this element of their wellbeing (Rizzo & Whitman 2009, 920 f.)? It cannot simply be presumed that individuals always and under any circumstances would choose what best promotes a specific end. As regards the pursuit of health, there are many other ends that can come in conflict with it. It is not straightforward whether people, say, want to eat salad more than a burger because it is a healthier option—even when it is agreed that they want to pursue a healthy diet.

## Which Choices Enhance Health?

Let us assume that people really want to choose a healthy option at more or less every occasion. That is, we assume that they are prepared to bear relevant costs, for instance foregoing an enjoyable experience for the sake of improving their health. In this section I want to argue that even if we make such a fairly implausible conjecture, there are still serious epistemic issues when introducing reasonable health-enhancing nudges. These problems are mainly due to confusions regarding the notion of health and connected issues to determine healthier options.

People normally want to be healthy in the sense that they do not want to have a disease. That is, they pursue health in the negative (or minimal) interpretation of absence of health.^9^ But, of course, every single option that is offered, say, in a canteen, is healthy in this respect. If any food option were to potentially cause a disease, it would be banned. In contrast, when we talk of healthy and unhealthy options, we normally interpret the relevant notion in a comparative sense. In terms of the example used before, a burger is not unhealthy in the sense of causing disease, but in the sense of being less healthy than a salad. The salad is less fatty, it contains more vitamins and minerals, and it has fewer calories. Accordingly, we switch to a gradual notion of health, where certain options can be compared in terms of their contribution to health in the sense of a lower risk of falling ill or developing a disease.

This second notion of health is not completely remote from the minimal (absolute) notion of health as absence of disease. Still, *health* is used in a different meaning than before; perhaps a better term would be *fitness*. Fitness is a dispositional condition of a person that makes is less likely—along a spectrum of propensity—to fall ill. By using the alternative term, it can easily be seen that the presumption regarding the relevant attitudes of people is far more dubious than it seemed: Although probably all people want to be healthy, not so many people want to enhance their fitness—given the costs—and even fewer people want to be fitter than they are already.

It might be objected that the relevant aims of health-enhancing nudges are actually in line with traditional notions of disease prevention. In other words, they might be deemed to be focused on negative health, not positive health. Some conditions connected to obesity, for instance, are diseases and health-related nudges are aiming at preventing such pathological conditions, not at making people more perfect, as it were. But this objection fails: Even disregarding the tenuous causal relationship between individual choices and disease dispositions, disease and health risks—or disease dispositions—are simply two separate types of conditions with different normative significance (cf. Schwartz, 2008). Risk of disease is not disease, and a good health disposition is not the same as a condition preventing disease. This has repercussions for assessing relevant interventions. Eating nutritious food is not comparable to, say, receiving a vaccine that directly prevents a specific disease.

Once we have a clear view on the actual aim of health-enhancing nudges being comparative fitness, not minimal health, we can also see that the epistemic problems multiply. This is because it is not at all clear which choices generally enhance fitness. Positive health, a condition over and above the absence of health, is reached by multiple pathways. It is well-known, for instance, that relaxation and a feeling of security can make it less likely to fall ill (Coupland, 2007). So-called comfort food can ease concerns and reduce stress-levels of people and hence contribute to their fitness, although it has to be acknowledged, of course, that eating—especially if done mindlessly—may well have negative fitness effects as well. Still, any calculation as to which choices will altogether enhance fitness will be extremely complicated, to say the least.

Advocates of health-enhancing nudges usually circumvent these epistemic problems by focusing only on one aspect of choices, usually nutritional values of different food options. This could be called the mechanistic aspect, because it relies on an explanation of health in terms of a well-functioning machine. Regarding the examples we have discussed, it can then be said that a burger is generally a less healthy option than a salad because its nutritional value makes it (if ever so slightly) more likely to develop diseases. But this does not settle the relevant questions, of course, because a burger might at least occasionally be more health-enhancing in a more holistic understanding of the term, which includes far more aspects than nutrition. What is more, even if the measure of healthy choices is reduced to, say, calorie intake—obviously a significant blinker to a holistic view on health—relevant empirical studies show no significant effects of tested health-enhancing nudges (Marlow, 2016; Freeman, 2016).^10^

Another common strategy to defend health-enhancing nudges is to highlight the benefits to population health, as opposed to individual health. Public health practitioners can argue that although eating comparatively more nutritious food might not always enhance the individual health of each citizen such consumption will still lead to an improvement of population health. This is a fair point, but again it relies on conceptual confusion between different conceptions of health; this time between individual health and population health. In terms of the potential efficacy of health-enhancing nudges, the population health perspective is of very restricted value. After all, epidemiology creates knowledge about incidences within populations, i.e. prevalence of disease, not about the causes of individual illness or fitness (see already Rose, 1985). In less complicated words: Public health researchers might find that groups of people who purchase more nutritious food are statistically less likely to be overweight, yet this data as such of course says nothing about the outcomes of health-enhancing nudges for individual choosers.

The epistemic problems in relation to health-enhancing nudges have led us to an important expansion of focus: Health should not be seen merely as a mechanistic or even a medical term, addressing only physiological and mental functioning. Once we expand our agenda in the right way, it is virtually impossible to make any general judgements regarding the comparative health-enhancing value of different choices. Indeed, this result seems hardly surprising, because we intuitively know that improving health and wellbeing is a formidable task, requiring constant adaptation to changing circumstances. Individual people are usually pretty good at navigating through their life in the way that they deem best. They can make individual assessments regarding different health-related values, such as nutrition, pleasure, quality of personal relationships, leisure etc. For instance, they might join a group of friends going out for a couple of drinks and pizza, and, on the next day at work, forego the burger and eat a salad instead. It is not straightforward why we would want to assign planners, let alone governmental nudging agencies, to such a formidable task of helping us to navigate through our lives. Nudgers’ epistemic limitations are more severe than those of people who live their own individual lives, although the latter are not always perfect in their judgement, of course. The complexities of life cannot be wrapped up in a meal choice.

To be sure, we want to help people avoiding serious mistakes, and that might imply helping them to avoid unhealthy choices. Yet this only takes us as far as common health and safety regulations are concerned, for instance banning toxic ingredients. In addition, the mentioned aim of avoiding serious mistakes also requires certain competences to be installed in people, especially a certain level of what is called health literacy (Kickbusch, 2009) and some conscious debiasing (Bovens, [2009], 213; Barton and Grüne-Yanoff, 2015). Avoiding less healthy choices also requires regulation of potentially fraudulent claims by the advertising industry or weight-loss companies to enable relevantly informed choices. But helping people to avoid relevant harm to self does not justify health-enhancing nudges.^11^ Altogether, the epistemic problems, regarding the individual value of health and regarding the effectiveness of specific choices in relation to their contribution to fitness, underline the conclusion that health-enhancing nudges will usually fail to reach their ambitious goal of enhancing health or fitness.^12^

Defenders of nudging can perhaps stress the point that health-enhancing nudges are nevertheless a better option than doing nothing or leaving the design of choices to interested parties, such as the food industry. In other words, health-enhancing nudges might be comparatively better than no nudges or malevolent nudges. In the following section of the paper, I aim to show that this defence of health-enhancing nudges fails, because—in a slogan—being paternalistically nudged is actually often bad for our health.

## Health-Enhancing Nudges can be Bad for Your Health

Many critics of nudges have claimed that nudges undermine individual autonomy and therefore pose a moral problem (see, e.g., Hausman and Welch, [2010], Rebonato, [2012], Wilkinson, 2013; White, 2013). In contrast, defenders have pointed out that nudges significantly differ from cases of manipulation and coercion and hence maintain personal autonomy (Hanna, 2015; Noggle, 2018; Mills, [2015], 497). After all, nudges leave all options intact, they do not close any doors, to use a widely used metaphor. Some additionally argue that although nudges can be manipulative this does not mean that they are therefore unjustified (Eyal, 2016). Such a quarrel cannot be decided unless we have a full analysis of the notion of individual autonomy and its moral relevance, which is obviously beyond the scope of this paper. If we start, however, from a very basic distinction between autonomy as a success-term and as a performance-term, we can see that autonomy can be undermined even if people reach their goals. We will also be able to discuss why specifically health-enhancing nudges are morally dubious.

It is true that nudges are compatible with autonomy, if we understand the term as succeeding in getting what one wants or prefers (Engelen & Nys, [2020], 144 f.).^13^ If individuals do not agree with the aim of nudges, determined choosers can easily dodge the impact of nudges. What is more, insofar as nudges are really helping people to overcome mistakes or to achieve what they really want, they can even be deemed autonomy-enhancing (Sunstein, 2016a, 51 ff.; Sunstein, 2016b, 65; Hallsworth, [2017], 47).^14^ Accordingly, if nudges threaten autonomy at all, it will have to be due to an impact on the process of will formation or decision making. I believe that such an impact on the performance of autonomy is indeed posing a real concern; a concern, that is usually disregarded by proponents of nudges. In the helpful phrase of Seana Shiffrin, nudges can manifest an “intrusion into and insult to a person’s range of agency” (Shiffrin, [2000], 218).^15^

I claim that the very fact that someone intentionally fiddles with the circumstances of choice in order to improve decisions regularly leads to resentment—not in all people, of course, but in a considerable number of people.^16^ This leads us back to the initial claim of proponents of nudges according to which people make mistakes that should be straightened out. The message of nudges is that people should not have sovereignty, even as regards the intricacies of personal life—such as making a meal choice in the canteen or observing drinking limit suggestions. This message annoys many people, I submit, and leads to a legitimate complaint regarding health-enhancing nudges. This complaint is based on a desire to being left to one’s own devices; it is concerned with having one’s own sphere; with being a sufficiently capable agent; not with the specific mechanisms or results of nudges. The implicit and occasionally explicit assumption of inferior agency only adds insult to injury.^17^

Consider Jeremy Waldron’s rhetorical question: “What becomes of the self-respect we invest in our own willed actions, flawed and misguided though they often are, when so many of our choices are manipulated to promote what someone else sees (perhaps rightly) as our best interest? “(Waldron, [2014], 22) Even if we agree with their advocates that nudges are different from full-fledged manipulation, there is a nagging and legitimate concern about the very intention to nudge people to health, wealth or happiness, and the message it sends. Human beings are agents and that involves being choosers. Even an imperfect chooser deserves to be fully recognised.^18^ Being nudged can lead to legitimate upset and often results in resentment. Now, we know that resentment, stress, anger, and similar negative feelings are bad for our physical and mental health, in the sense of making it more likely to develop certain medical disorders, such as depression or heart disease.^19^ Accordingly, being nudged can be bad for our health.

Health-enhancing nudges belong to an area of personal life that many people have strong feelings about. Food and drink choices are important to people, and there are significant cultural aspects regarding eating and drinking habits. In other terms, such choices concern the value of people’s food-related experiences and identities (Barnhill et al. 2014; cf. White, [2016], 76). It is hence to be expected—though admittedly it would require some empirical study to confirm—that health-enhancing nudges are particularly resented. Therefore, rather paradoxical, health-enhancing nudges can easily be bad for (some) people’s health.

It can be countered that governmental nudges are publicly induced and usually democratically legitimised, if only indirectly (Gingerich, 2016).^20^ People who are unhappy with health-enhancing nudges are free to organise opposition; otherwise they simply ought to succumb to decisions to introduce nudges, legitimised by democratically elected leaders. Indeed, this is a conclusion that applies to almost every political decision. In response, it is true, of course, that public regulations can never make everyone happy. However, I believe the argument of this paper has established that we should not be too cavalier about health-enhancing nudges. Quite a few citizens really do not want them for good reasons, and those who agree with nudges might be swayed in their opinion once they learn that health-enhancing nudges are actually inadequate.

## Conclusion

I have argued that planned health-enhancing nudges fail to fulfil their supposed function. The main problem I have identified is the complexity of the overarching value, health, itself. This poses an epistemological problem in addition to the knowledge problems that have been discussed in the relevant literature (cf. Qizilbash, 2012; Sugden, [2008], 232). I have further argued that health-enhancing nudges are liable to resentment. This argument was put forward in relation to an assumed element of the good life for human beings—being a chooser and agent of one’s own life. Advocates of nudges promote different interpretations of basic values. Sunstein, for instance, is mainly interested in a reduced understanding of health as normal organismic functioning and welfare as including measurable elements, for instance financial means.

In the end, it seems to me, we have to make a choice whether we want to live in a planned and designed perfectionist world aiming at health-enhancing choice, or in the messy, often failing, but perhaps more humane world of personal agency, where health is just one element of a complex life.

## Footnotes

1 I agree with Andreas Schmidt and Bart Engelen, who conclude in their recent overview that “there is an increasing understanding that ethical concerns differ radically from practice to practice and from nudge to nudge” and that hence “ethical analysis should proceed case by case” (Schmidt & Engelen, [2019], 9).

2 I avoid the commonly used term *choice architecture*, because other people might represent mechanisms of nudging—say, when role models are intentionally introduced—and they are hardly part of what would normally be called an architecture, though they are part of the environment of a chooser. Some scholars define nudges as mechanisms that intentionally use the same psychological imperfections (biases etc.) that seem to make nudges necessary in the first place (White, [2016], 21). I do not think nudges necessarily work on non-rational psychological mechanisms.

3 It is true that Thaler and Sunstein, in one of their first publications on the topic, were mainly preoccupied with paternalistic nudges. Yet in their book *Nudge*, first published in 2008, they more generally consider nudges as “any factor that significantly alters the behavior in a predictable way without forbidding any options or significantly changing their economic incentives” (Thaler & Sunstein, [2008], 9). This allows for paternalistic and non-paternalistic goals. Some of their examples accordingly target externalities, for instance a proposed “civility check” to avoid impolite emails or the programme “dollar a day” to reduce teenage pregnancy. The latter is advised because “taxpayers end up paying a significant amount for many children born to teenagers” (ibid. 232 f.). Gigerenzer (2015, 363) recommends a more restrictive conceptualisation, which only allows for paternalistic nudges.

4 Sunstein goes on to call the use of a GPS device a form of so-called means paternalism (Sunstein, [2020], 44), which aims at making the choice of the best means for a set end more likely. However, such a perspective additionally makes the notion of paternalism far too broad. In an earlier study, Sunstein even claimed that “[n]ature itself nudges; so does the weather” (Sunstein, 2016a, 21). This, for him, is then evidence that it is “pointless to object to choice architecture or nudging as such” (ibid.). He calls this his “first central argument” in favour of governmental nudges (ibid., 23).

5 In an elaborate analysis of nudges, Christopher McCrudden and Jeff King deem unhealthy or risky lifestyles harmful to others instead of merely self-regarding. For them, “pensions, cigarettes and obesity” are related to forms of “risk-taking [that] is in reality free-riding on the generosity of those who pick up the pieces after the gamble is lost” (McCrudden & King, [2016], 102). I do not take a stance towards such an interpretation of imperfect health-related behaviour as harmful to others.

6 Personal nudges are not my main concern. Intuitively, they seem reasonable, because they are a form of self-binding or externally reinforced commitment.

7 In a recent publication, Sunstein refers to what choosers themselves value in the sense of an ideal, modelled chooser (Sunstein, [2020], 60). Similarly, he believes that planners need to make assumptions about the “best understanding of welfare” (ibid., 38).

8 Sunstein and Thaler call real individuals that are subject to psychological mechanisms, such as biases, “Humans” and idealised choosers “Econs” (Thaler & Sunstein, [2008], 7 ff.). Econs are based on the traditional *homo economicus* model, as developed by neoclassical economics. Behavioral economists reject that model and aim at a psychologically more realistic analysis. Given their own idealisations, I believe Sugden is right when he says that Sunstein and Thaler actually do not discuss real humans but faulty Econs (Sugden, [2017], 117; see also Rizzo, [2020], 46 ff.).

9 The debate on the concept of health in philosophy of medicine is fairly advanced. Helpful introductions are Nordenfelt, [2017] and Schramme, [2019], 17ff.

10 One recent survey found that the most effective nudges reduced daily calorie intake by 209 kcal (Cadario et al. 2018, 29). Whether there was any long-term effect on calorie-intake over a period of time was not tested. For comparison, the National Health Service of the UK recommends a daily calorie intake of 2,000 kcal a day for women and 2,500 kcal for men (https://www.nhs.uk/common-health-questions/food-and-diet/what-should-my-daily-intake-of-calories-be/). There are numerous other reviews of effectiveness, which seem to support a more optimistic outlook. Yet, these usually measure the impact of nudges on consumption choices, which is obviously different from impact on health (see, e.g., Adam and Jensen, 2016; Mertens et al., 2022). In fairness, there are studies that explicitly acknowledge the need for outcome measures (Bucher et al., 2016).

11 It is interesting to see that a recent trend in behavioral economics indeed pushes against individual nudges and for system-oriented interventions, such as traditional legal frameworks, tackling discrimination and economic destitution, as well as education (Chater & Loewenstein, 2022).

12 Self-induced (personal) health-enhancing nudges might fare better, but they also cannot solve the epistemic problems surrounding the task of identifying holistically effective choices.

13 Engelen and Nys [2020] use the term “autocracy” for this interpretation of autonomy, thereby following Paul Guyer’s formulation.

14 Sunstein claims that philosophers’ understanding of autonomy is somewhat limited anyway: “[I]n speaking of autonomy, philosophers could learn a great deal from economists, who know that the real question is usually not whether people are going to be allowed to make choices, but whether someone (such as government) will or should impose costs on people who make choices” (Sunstein, 2016b, 63).

15 In contrast to philosophers who discuss the manipulative nature of nudges and see them perhaps as a kind of “usurpation of will” (Dworkin, [1983], 107), I am more interested in the second part of Shiffrin’s description, the relevant insult that comes with assuming better agency than the nudged person.

16 This is an empirical claim, mainly based on anecdotal evidence. I do not think, though, that it is a far-fetched assumption. For some empirical evidence supporting my claim see, e.g., Thunström, [2019]. The negative effects of nudges are here fleshed out in terms of an emotional tax.

17 It is true, of course, that such kind of insult is not specific to nudges, but other interferences as well. Still, it applies here.

18 Surely there are limits to this freedom. Importantly, the potential impact of a choice, for instance if it is life-threatening, makes a difference. But these are not the cases we discuss in this paper, of course.

19 Admittedly, I am not an expert regarding the medical impact of negative emotions, but there is empirical evidence supporting my assumption (Smith et al. 2006; Novaco 2010).

20 I ignore the issue of nudges being usually covert—undisclosed to nudged individuals—and hence more difficult to control democratically. One study suggests that transparency about nudges does not alter their effects, at least as tested in a particular scenario regarding default options about hypothetical advance directives (Loewenstein et al., 2015). Interestingly, Sunstein himself claims that all nudges should be transparent (Sunstein, [2020], 55 f.). By that, he seems to mean that they should be democratically controlled—“[n]udges should be visible, scrutinized, and monitored” (ibid., 55)—not that they should be made conscious to individual choosers.
